# Prediction of Left Atrial Fibrosis and Success of Catheter Ablation by Speckle Tracking Echocardiography in Patients Imaged in Persistent Atrial Fibrillation

**DOI:** 10.3389/fcvm.2022.856796

**Published:** 2022-05-25

**Authors:** Sébastien Marchandise, Quentin Garnir, Christophe Scavée, Varnavas Varnavas, Jean-Benoit le Polain de Waroux, Aurélien Wauters, Christophe Beauloye, Véronique Roelants, Bernhard L. Gerber

**Affiliations:** Division of Cardiology, Department of Cardiovascular Diseases, Cliniques Universitaires St. Luc, Pôle de Recherche Cardiovasculaire (CARD), Institut de Recherche Expérimentale et Clinique (IREC), Université catholique de Louvain, Brussels, Belgium

**Keywords:** persistent atrial fibrillation, speckle tracking echocardiography, strain, atrial scar, catheter ablation – atrial fibrillation, atrial fibrillation

## Abstract

**Background:**

Non-invasive evaluation of left atrial structural and functional remodeling should be considered in all patients with persistent atrial fibrillation (AF) to optimal management. Speckle tracking echocardiography (STE) has been shown to predict AF recurrence after catheter ablation; however in most studies, patients had paroxysmal AF, and STE was performed while patients were in sinus rhythm.

**Aim:**

The aim of this study was to evaluate the ability of STE parameters acquired during persistent AF to assess atrial fibrosis measured by low voltage area, and to predict maintenance of sinus rhythm of catheter ablation.

**Methods:**

A total of 94 patients (69 men, 65 ± 9 years) with persistent AF prospectively underwent measurement of Global Peak Atrial Longitudinal Strain (GPALS), indexed LA Volume (LAVI), E/e′ ratio, and LA stiffness index (the ratio of E/e′ to GPALS) by STE prior to catheter ablation, while in AF. Low-voltage area (LVA) was assessed by electro-anatomical mapping and categorized into absent, moderate (>0 to <15%), and high (≥15%) atrial extent. AF recurrence was evaluated after 3 months of blanking.

**Results:**

Multivariable regression showed that LAVI, GPALS, and LA stiffness independently predicted LVA extent after correcting for age, glomerular filtration rate, and CHA_2_DS_2_-VAS_c_ score. Of all the parameters, LA stiffness index had the highest diagnostic accuracy (AUC 0.85), allowing using a cut-off value ≥0.7 to predict moderate or high LVA with 88% sensitivity and 47% specificity, respectively. In multivariable Cox analysis, both GPALS and LA stiffness were able to significantly improve the c statistic to predict AF recurrence (*n* = 40 over 9 months FU) over CHARGE-AF (*p* < 0.001 for GPALS and *p* = 0.01 for LA stiffness) or CHA_2_DS_2_-VAS_c_ score (*p* < 0.001 for GPALS and *p* = 0.02 for LA stiffness). GPALS and LA stiffness also improved the net reclassification index (NRI) over the CHARGE-AF index (NRI 0.67, 95% CI [0.33–1.13] for GPALS and NRI 0.73, 95% CI [0.12–0.91] for LA stiffness, respectively), and over the CHA_2_DS_2_-VAS_c_ score (NRI 0.43, 95% CI [−0.14 to 0.69] for GPALS and NRI 0.52, 95% CI [0.10–0.84], respectively) for LA stiffness to predict AF recurrence at 9 months.

**Conclusion:**

STE parameters acquired during AF allow prediction of LVA extent and AF recurrence in patients with persistent AF undergoing catheter ablation. Therefore, STE could be a valuable approach to select candidates for catheter ablation.

## Introduction

The management of patients with persistent atrial fibrillation (AF) remains challenging. AF catheter ablation (CA) is recommended for rhythm control to improve symptoms of AF recurrence after failed antiarrhythmic drug therapy and may also be considered as first-line therapy in patients without risk factors for recurrence (1). However, CA is less successful in persistent than in paroxysmal AF (2) and its success is predicted by many factors, among which structural atrial remodeling and atrial scar burden (3) are currently considered the mainstay for AF recurrence (4, 5). Therefore, the decision of whether to pursue CA in persistent AF should be based on considering the risk factors for AF recurrence following the procedure and, in particular, atrial scar burden.

The atrial scar can be assessed by several modalities (6, 7). Cardiovascular magnetic resonance imaging with the late enhancement of gadolinium (LGE-cMR) has been the most studied (8). However, it was found to have no added value to guide ablation strategy (9) and its complexity and limited accessibility restrict its use in clinical practice. LA scar extent may be also evaluated by low-voltage area (LVA) on electroanatomical mapping (EAM) performed during the CA procedure. Several studies showed that extensive LVA extent predicts increased post-ablation arrhythmic recurrences (10, 11). However, because EAM is only available at the time of ablation, this modality is not of value for the selection of CA candidates.

Echocardiography with speckle tracking (STE) allows quantitative assessment of LA volume, LA pressure, LA function, and estimates of LA stiffness could present a non-invasive approach to evaluate atrial structural remodeling. Indeed, LA strain and strain rate were found to correlate with LA fibrosis by LGE-cMR (12) and to be associated with the risk of AF and stroke. STE LA strain was also shown to predict the recurrence of atrial arrhythmias after CA or electrical cardioversion (13–18). However, most studies evaluated STE, while patients were in sinus rhythm (SR) at the time of echocardiographic analysis. Therefore, in this work, we aimed at evaluating the value of STE in homogeneous population of patients being in persistent AF at the time of analysis to assess atrial scar burden by EAM-LVA and to predict the success of CA.

## Materials and Methods

### Study Design and Population

Between January 2018 and April 2021, we prospectively enrolled patients after providing written informed consent to the Institutional Review Board (IRB) approved (2018-17OCT-389) study protocol (TRIATLON, EudraCT 2019-001813-17A). Inclusion criteria were adult patients with persistent non-valvular AF referred for a first CA procedure according to the ESC guidelines (1) who had preprocedural transthoracic echocardiography (TTE) less than 2 months before undergoing the procedure while being in persistent AF. Patients with a history of congenital heart disease, valvular disease, genetic heart diseases, previous ablation procedure, or those with left atrial appendage thrombus identified at the preoperative transesophageal echocardiography contraindicating CA were not considered for inclusion.

### Clinical Parameters

All patients underwent clinical examination, ECG, and comprehensive laboratory testing, including full blood count, and evaluation of renal, hepatic, and thyroid function prior to CA. We prospectively recorded coefficient of variation (CV) risk factors, duration of AF, known obstructive sleep apnea, and computed CHA_2_DS_2_-VASc, HATCH, and APPLE risk scores for recurrence of AF as well as HAS-BLED score for risk of bleeding. Symptom severity was classified with EHRA score.

### Echocardiography

Pre-intervention two-dimensional (2D) TTE was acquired while patients were in AF using IE33 or EPIC echocardiographic systems (Philips Healthcare, Andover, MA, United States) and stored digitally on a PACS system (Philips Intellispace). Images were analyzed offline by one observer (SMA) and all measurements were performed by averaging 2 cardiac cycles. Left ventricular (LV) volumes and ejection fraction were calculated using Simpson’s biplane method. The E/e′ ratio was calculated by the dividing peak mitral flow E wave by the average of septal and lateral e’ tissue Doppler velocities.

LA volume and strain analysis were performed on dedicated apical 4 and 2-chamber views of the LA acquired with a frame rate between 60 and 80 frames per second and analyzed using Image-Arena™ cv4.6 (TomTec Imaging Systems, Unterschleissheim, Germany) in duplicate by two experienced blinded echocardiographers (SMA and QG). The LA endocardium was traced automatically with manual adjustment if needed. According to the recommendation of the EACVI/ASE Task Force, Reservoir Global Peak Atrial Longitudinal Strain (GPALS) was computed using LV end-diastole as zero-strain point. Maximal and minimal LA volume was measured, respectively, at the end of LV systole and at the end of the LV diastole and indexed to the body surface area (LAVI). LA fractional area change (FAC) and ejection fraction (LAEF) of passive emptying fraction were computed, respectively, as the percentage ratio of change of LA area and volume during systole. LA stiffness index (LASI) was calculated as E/e′ divided by GPALS (19).

### Electroanatomical Mapping and Catheter Ablation

**FIGURE 1:**
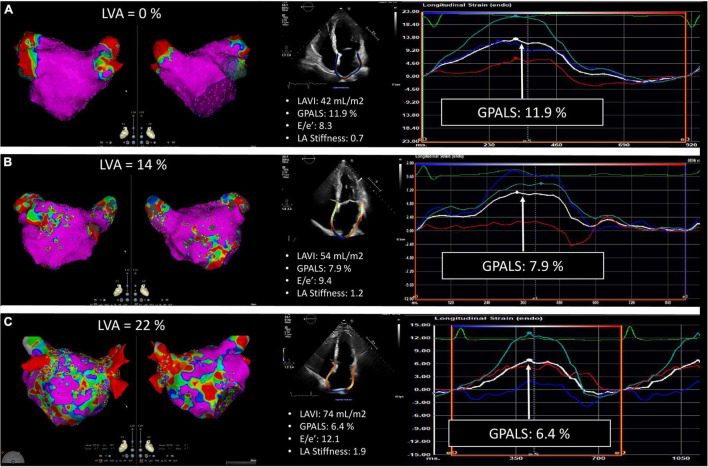
Representative example of echocardiographic strain analysis performed in three patients with different severity of left atrial (LA), low-voltage area (LVA) extent. Group I: no LVA **(A)**, group II: moderate LVA extent **(B)**, and group III: important LVA extent **(C)**. The color gradient of the LVA maps indicates electrogram amplitude from pink (>0.5 mV in SR and >0.31 in AF) to red at <0.1 mV.

Electrical cardioversion was attempted at the beginning of the procedure prior to ablation. EAM of the LA was obtained using the ConfiDENSE™ CARTO3 system v6.0.70 using the 2515 Variable Loop Lasso Multi-Electrode Eco Nav (Biosense Webster, Diamond Bar, CA, United States) using at least 1,000 measurements. LVA was defined as bipolar peak-to-peak voltage amplitude of <0.5 mV in SR and <0.31 mV in AF. The total LVA extent was calculated as the percentage of LA surface and patients were separated into 3 groups of LVA extent: Group I (absent): LVA = 0%, Group II (moderate): LVA >0 and ≤15%, and Group III (high): LVA >15% (Figure 1). All patients underwent radiofrequency PVI. Additional lines or complex atrial fractionated electrogram (CAFE) ablations were performed at the physician’s discretion.

### Clinical Follow-up

Patients underwent clinical follow-up and ECG at 3 months, 6 months, and 1 year as well as 24-h ambulatory Holter monitoring at 6 months after CA. Additionally, surface ECG, ambulatory ECG, and/or cardiac event recording values were obtained when patients presented symptoms of arrhythmia recurrence. Any detectable atrial tachyarrhythmia (AF, atrial flutter, or atrial tachycardia) of at least 30 s beyond a 3-month blanking period after the procedure was considered as recurrence.

### Statistical Analysis

Statistical analysis was performed using SPSS version 26.0 (SPSS, Inc., Chicago, IL, United States) and R 3.5. Continuous values were tested for normality by Q-Q plots, histograms, and the Kolmogorov–Smirnov test. Normally and non-normally distributed baseline parameters between different LVA stages were expressed as the mean ± SD and median and interquartile range (IQR) and were compared, respectively, using one-way ANOVA or Kruskal–Wallis test when not normally distributed. *Post hoc* comparison between groups was performed using Tukey’s test and the Wilcoxon rank-sum test, as appropriate. Categorical data were compared using the Chi-square test. A *p*-value < 0.05 was considered indicative of a statistically significant difference. The relationship between LVA and echocardiographic parameters was evaluated by linear regression analysis and the Pearson correlation coefficient. Uni- and multivariable binary logistic regression analyses were used to determine associative factors predicting moderate (Group II) or important (Group III) LVA extent, respectively. Parameters with significant collinearity were not allowed to simultaneously enter the multivariable model. Collinearity was evaluated by multiple regression analysis between parameters and we considered evident collinearity when tolerance (1−*R*^2^) was <0.2 or variance inflation factor >5. Receiver operating characteristic (ROC) analysis was performed using the roc.test function in the pROC package, and areas under ROC curves were compared pairwise using the Delong test. Optimal sensitivity and specificity were determined by the highest Youden index. Recurrence-free survival was related to the individual clinical parameters, LVA extent, and echocardiographic covariables, and was evaluated using Kaplan–Meier survival graphs and uni- and multivariable Cox proportional hazard regression models. We also evaluated the incremental benefit of GPALS and LA stiffness, to improve the prediction of AF recurrence over either CHARGE-AF or CHA_2_DS_2_-VAS_c_ score by computing the increase in the c statistic in the Cox model and by computing their net reclassification index (NRI).

Intraobserver and interobserver reproducibility of strain measurements were assessed in 10 randomly selected patients and expressed as CV and intraclass correlation coefficient (ICC) with 95% confidence interval. The CV was expressed as a percentage (mean/SD).

## Results

### Patient Characteristics

Patient baseline characteristics are summarized in Table 1. A total of 94 patients (69 men, mean age 65 ± 9 years) were included. AF persisted for <1 year in 86 (91%) patients and for >1 year in 8 (9%) patients. Also, 72% of patients were under antiarrhythmic medications. Mean CHA_2_DS_2_-VASc score was 2.3 ± 1.6 and 11 (12%) patients had a history of stroke/TIA or systemic thromboembolism. Notably, 51 patients (54%) were hypertensive, 13 (14%) had coronary artery disease, 11 (12%) had diabetes mellitus, and 15 (16%) had sleep apnea.

**TABLE 1:** Study population.

	All *n* = 94	LVA Group I (0% LVA) *n* = 38	LVA Group II (LVA 0–15%) *n* = 37	LVA Group III (LVA >15%) *n* = 19	*p*-Value
**Clinical characteristics**					
Age (years)	65 ± 9	62 ± 9^III^	66 ± 8	69 ± 5	0.008
Sex (male)	69 (73%)	30 (79%)	29 (78%)	10 (53%)	0.07
BMI (kg/m^2^)	29 ± 5	29 ± 5	29 ± 5	28 ± 5	0.62
Hypertension	50 (53%)	17 (45%)	20 (54%)	13 (68%)	0.24
Hypercholesterolemia	46 (49%)	20 (53%)	15 (41%)	11 (56%)	0.40
Diabetes	11 (12%)	5 (13%)	4 (11%)	2 (10%)	0.94
Family history of CAD	27 (29%)	14 (37%)	13 (35%)	7 (37%)	0.16
Smoking	29 (30%)	14 (37%)^III^	13 (35%)^III^	1 (5%)	0.03
CAD	13 (14%)	5 (13%)	5 (13%)	3 (16%)	0.49
Chronic lung disease	19 (20%)	10 (26%)	5 (13%)	4 (21%)	0.39
Sleep apnea	15 (16%)	6 (16%)	7 (19%)	2 (11%)	0.42
Systolic blood pressure (mmHg)	129 ± 16	131 ± 15	128 ± 17	128 ± 19	0.63
Diastolic blood pressure (mmHg)	79 ± 12	79 ± 13	77 ± 11	81 ± 10	0.54
Heart rate (bpm)	83 ± 20	81 ± 21	86 ± 20	82 ± 17	0.82
eGFR (ml/min)	76 ± 17	82 ± 15^III^	75 ± 17	66 ± 18	0.005
Stroke/TIA history	11 (12%)	6 (16%)	5 (14%)	3 (16%)	0.63
Vascular disease	15 (16%)	8 (21%)	4 (11%)	3 (16%)	0.49
AF duration >6 months	35 (37%)	11 (29%)	13 (35%)	11 (58%)	0.12
CHA_2_DS_2_-VASc score	2.3 ± 1.6	1.9 ± 1.3^III^	2.3 ± 1.7	3.2 ± 1.4	0.011
CHARGE-AF score	12.7 ± 1.0	12.4 ± 1.1	12.8 ± 0.9	13.0 ± 1.0	0.063
HATCH score	1.7 ± 1.3	1.4 ± 1.2^III^	1.7 ± 1.3^III^	2.5 ± 1.2	0.002
APPLE score	2.6 ± 1.0	2.2 ± 1.1^II,III^	2.6 ± 0.9	3.2 ± 1.0	0.006
HAS-BLED score	1.4 ± 1.1	1.1 ± 1.0^III^	1.4 ± 1.1^III^	2.1 ± 0.8	0.004
**EHRA score**					
I	15 (16%)	8 (21%)	6 (16%)	1 (5%)	0.59
II	47 (50%)	16 (42%)	20 (54%)	11 (58%)	
III	30 (32%)	13 (34%)	11 (30%)	6 (32%)	
IV	2 (2%)	1 (3%)	0 (0%)	1 (5%)	
**Medications before CA**					
β-Blockers	66 (70%)	24 (63%)	27 (73%)	15 (79%)	0.43
Sotalol	8 (9%)	5 (13%)	3 (8%)	0 (0%)	0.25
Amiodarone	20 (21%)	6 (16%)	10 (27%)	4 (21%)	0.50
AAR class I	18 (19%)	9 (21%)	6 (16%)	4 (21%)	0.77
Digoxin	4 (4%)	2 (5%)	2 (5%)	0 (0%)	0.60
**Echocardiography**					
LVEF (%)	56 ± 39	56 ± 16	55 ± 12	57 ± 14	0.88
LV mass index (g/m^2^)	93 ± 28	86 ± 22	100 ± 30	101 ± 29	0.08
LA diameter (mm)	44 ± 6	42 ± 6^II^	46 ± 5	46 ± 5	0.02
LAVI (ml/m^2^)	59 ± 19	50 ± 13^II,III^	63 ± 19^III^	64 ± 19	<0.001
E wave (cm/s)	80 ± 20	76 ± 16	80 ± 20	89 ± 22	0.07
Average e′ (cm/s)	9.5 ± 2.6	10.8 ± 2.5	9.0 ± 2.6	8.1 ± 1.4	0.002
E/e′	9.1 ± 3.2	7.9 ± 2.5^III^	9.0 ± 3.3^III^	11.4 ± 2.9	<0.001
LAEF (%)	19 ± 11	22 ± 14^III^	18 ± 6	15 ± 9	<0.001
LA-FAC (%)	14 ± 7	16 ± 8^III^	13 ± 4	11 ± 6	0.02
GPALS (%)	8.9 ± 4.5	11.3 ± 5.3^II,III^	7.9 ± 2.6	6.2 ± 2.9	<0.001
LA stiffness	1.4 ± 1.1	0.8 ± 0.4^III^	1.3 ± 0.8^III^	2.5 ± 1.6	<0.001

*AF, atrial fibrillation; AAD, anti-arrhythmic drugs; BMI, body mass index; EDV, left ventricular end diastolic volume; EF, ejection fraction; eGFR, estimated glomerular filtration rate; ESV, end systolic volume; FAC, left atrial fractional area change; GPALS, Global Peak Atrial Longitudinal Strain; LA, left atrium; LAVI, left atrial volume indexed; LV, left ventricular; TIA, transient ischemic attack. ^II^Post hoc comparison p < 0.05 vs. Group II. ^III^Post hoc comparison p < 0.05 vs. Group III.*

Electroanatomical mapping was successfully performed in 74 patients (79%) during AF and in 20 patients (21%) during SR (after cardioversion during CA procedure). LVA was observed in 56 (60%) patients with a median extent of 2.5% (range 0–64%). By EAM, 38 patients had no LVA (Group I), 37 patients had moderate LVA (Group II), and 19 patients had important LVA extent (Group III). The patients in the higher LVA groups were older, had lower estimated glomerular filtration rate (eGFR), and higher CHA_2_DS_2_-VASc, CHARGE-AF, and HAS-BLED scores. However, there was no difference in the coronary artery risk factors, AF duration, EHRA scores, or in antiarrhythmic drug therapy between the different groups.

### Catheter Ablation

The procedural characteristics are summarized in Table 2. PVI was successfully performed in all patients. Box isolation, other lines, and CAFE were performed more frequently in patients with higher LVA. There were no complications of cardiac perforation, systemic embolism or stroke, atrial-esophageal fistula, pulmonary stenosis, or death.

**TABLE 2:** Procedural characteristics.

	All *n* = 94	LVA Group I (0% LVA) *n* = 38	LVA Group II (LVA 0–15%) *n* = 37	LVA Group III (LVA >15%) *n* = 19	*p*-Value
LVA extent	8 ± 12	0 ± 0^II,III^	6 ± 4^III^	27 ± 15	*p* < 0.001
AF rhythm during EAM	74 (79%)	28 (73%)	30 (81%)	16 (84%)	0.11
PVI	94 (100%)	38 (100%)	37 (100%)	19 (100%)	NA
CTI	22 (23%)	12 (32%)	8 (22%)	2 (10%)	0.20
Posterior wall isolation	26 (28%)	1 (3%)^II,III^	12 (32%)^III^	13 (68%)	<0.001
Mitral isthmus	17 (18%)	1 (3%)^II,III^	7 (19%)^III^	9 (47%)	<0.001
CAFE	6 (6%)	0 (0%)^III^	2 (5%)	4 (21%)	0.01
Procedural time (min)	173 ± 42	162 ± 42	176 ± 39	188 ± 43	0.08

*AF, atrial fibrillation; CAFE, complex atrial fractionated electrogram; CTI, cavo-tricuspid isthmus ablation; EAM, electroanatomical mapping; LVA, low-voltage area; NA, not applicable; PVI, pulmonary vein isolation; TS, trans-septal puncture. ^II^Post hoc comparison p < 0.05 vs. Group II. ^III^Post hoc comparison p < 0.05 vs. Group III.*

### Echocardiographic Parameters and Relation to Low-Voltage Area Extent

**FIGURE 2:**
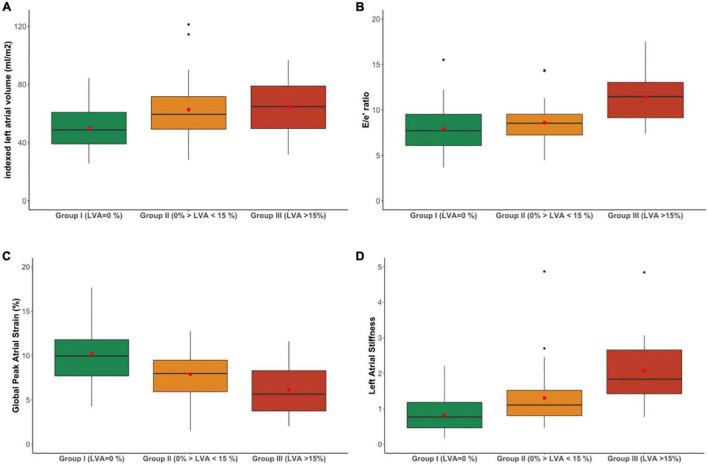
Indexed left atrial volume **(A)**, E/e′ ratio **(B)**, Global Peak Atrial Longitudinal Strain (GPALS) **(C)**, and left atrial stiffness **(D)** in patients with permanent AF according to their stages of low-voltage area extent. The box represents interquartile range, the line indicates the median value, whiskers show minimum/maximum values, and the mean value is shown as a red dot and outliers as black dots.

Table 1 summarizes the baseline 2D echocardiographic and STE findings. The patients in the moderate or high LVA extent groups had a significantly larger indexed LA volume (Figure 2A), higher E, and lower e′ wave velocities and thus higher E/e′ ratio (Figure 2B), lower LA ejection fraction, FAC and GPALS (Figure 2C), and greater LA stiffness (Figure 2D). However, there was no difference in the LV ejection fraction or LV mass index between the three groups of patients. LVA extent correlated significantly but poorly with LAVI (*r* = 0.20, *p* > 0.001) and better with GPALS (*r* = 0.38, *p* > 0.001), E/e′ ratio (*r* = 0.39, *p* > 0.001), and especially with LA stiffness (*r* = 0.49, *p* > 0.001).

By logistic regression analysis, the presence of LVA was significantly associated with age, eGFR, diuretics use, and with CHA_2_DS_2_-VASc, HAS-BLED, and CHARGE-AF risk scores (Table 3). Because of the association between GPALS, E/e′ ratio and LA stiffness, multivariable analysis was presented as two models, including either E/e′ and GPALS or LA stiffness. Multivariable analysis demonstrated that LAVI, and either GPALS or LA stiffness remained independently associated with presence of LVA after correcting for clinical parameters, such as age, eGFR, and CHA_2_DS_2_-VASc score (Table 3).

**TABLE 3:** Uni- and multivariable logistic regression analysis for predictors of LVA.

	Univariable analysis		Multivariable model 1		Multivariable model 2	
			
	OR [95% CI]	*p*-Value	OR [95% CI]	*p*-Value	OR [95% CI]	*p*-Value
Age	1.08 [1.02; 1.14]	0.007	0.99 [0.89; 1.09]	0.848	1.00 [0.91; 1.10]	0.945
Sex	1.07 [1.02; 1.13]	0.32				
BMI	1.63 [0.62; 4.29]	0.65				
Diabetes	0.97 [0.89; 1.07]	0.72				
Hypertension	1.26 [0.35; 4.47]	0.18				
CHARGE-AF risk score	0.56 [0.24; 1.29]	0.03				
CAD	0.48 [0.18; 1.24]	0.85				
Pulmonary disease	0.89 [0.26; 2.96]	0.23				
eGFR	1.86 [0.67; 5.14]	0.009	0.96 [0.92; 1.00]	0.06	0.96 [0.93; 1.00]	0.078
ARB/ACEI	0.96 [0.93; 0.99]	0.06				
Loop diuretics	0.36 [0.13; 1.02]	0.03				
AAR	0.29 [0.09; 0.87]	0.09				
CHA2DS2-VASC score	0.16 [0.01; 1.35]	0.03	1.11 [0.68; 1.79]	0.671	1.07 [0.66; 1.75]	0.763
HAS-BLED score	1.36 [1.02; 1.81]	0.02				
AF duration	1.62 [1.06; 2.47]	0.87				
Total # of episodes of AF	0.86 [0.67; 1.17]	0.29				
LVEF (%)	0.99 [0.96; 1.03]	0.90				
LV mass index	1.01 [0.99; 1.04]	0.11				
LAVI	1.05 [1.02; 1.09]	0.001	1.06 [1.01; 1.11]	0.016	1.06 [1.01; 1.11]	0.011
E wave	1.02 [0.99; 1.04]	0.09				
e′ wave	0.66 [0.50; 0.88]	0.005				
E/e′	1.30 [1.06; 1.59]	0.009	1.21 [0.95; 1.55]	0.115		
LAEF	0.95 [0.91; 0.99]	0.037				
FAC	0.91 [0.84; 0.98]	0.012				
GPALS	0.73 [0.62; 0.86]	<0.001	0.78 [0.64; 0.96]	0.02		
LA stiffness	8.24 [2.52; 26.9]	<0.001			4.97 [1.34; 18.4]	0.016

*OR, odds ratio, all others as Table 1.*

**FIGURE 3:**
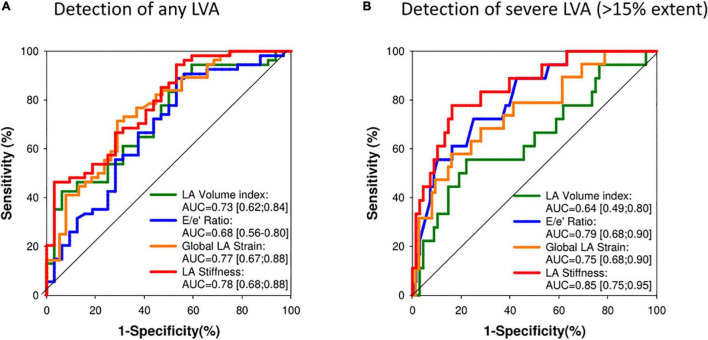
**(A)** Receiver-operating characteristic curve analysis for prediction of presence of any LVA during EAM (Groups II and III). AUC, area under the curve; *p* < 0.05 Global LA strain vs. E/e′ ratio. **(B)** Receiver-operating characteristic curve analysis for prediction presence of important LVA extent (Group III LVA >15%) during electroanatomical mapping (EAM). AUC, area under the curve; *p* < 0.05 LA strain vs. LAVI.

The ROC analysis for prediction of any (>0%) and important (>15%, Group III) LVA extent is shown in Figures 3A,B, respectively. LA stiffness had the highest AUC (0.78), for prediction of overall LVA extent, which was significantly (*p* < 0.05) greater than that of E/e′ ratio. Also, for prediction of important LVA extent, LA stiffness had the highest AUC (0.85), which was significantly higher than that of LAVI (AUC 0.64, *p* = 0.03) and GPALS (AUC 0.75, *p* = 0.01). A cut-off value of GPALS ≤10% allowed to predict moderate or important LVA with a sensitivity of 86 and 53% specificity, respectively, while LA stiffness ≥0.7 had 88% sensitivity and 47% specificity to predict important LVA.

### Prediction of Catheter Ablation Success Rate

After CA procedure, 60 patients (67%) were discharged with beta-blockers, 10 patients (11%) with sotalol, 24 patients (26%) with amiodarone, and 20 patients (21%) with class I AAR drugs. During a median of 9 [6; 11] months of follow-up, 40 (43%) patients had arrhythmia recurrence. Patients with recurrent atrial tachyarrhythmias were older (68 ± 6 vs. 64 ± 10 years, *p* = 0.009), more often hypertensive (68% vs. 43%, *p* = 0.02), and had a higher HAS-BLED score. By contrast, there were no differences in the CHA_2_DS_2_-VASc (2.6 ± 1.3 vs. 2.1 ± 1.8, *p* = 0.11), HATCH (1.7 ± 1.1 vs. 0.35 ± 1.34, *p* = 0.47), and APPLE risk scores (2.6 ± 0.9 vs. 2.5 ± 1.1, *p* = 0.68), or according to antiarrhythmic drug treatment administered after CA procedure [20 patients (49%) vs. 27 patients (51%), *p* = 0.55) between patients with and without AF recurrence. Patients with AF recurrence had however significantly lower GPALS (7.6 ± 3.8% vs. 9.9 ± 4.6%, *p* = 0.007) and greater LA stiffness (1.7 ± 1.3 vs. 1.1 ± 0.9, *p* = 0.04). Although LVA extent was greater in patients with recurrent atrial tachyarrhythmias (median 6.3; IQR 14.6) than in those with successful CA (median 0.7; IQR 8.7, *p* = 0.05), there were no significant differences (*p* = 0.35) of AF recurrence between patients with no (34%), moderate (46%), and high (53%) LVA.

Uni- and multivariable Cox regression analysis of AF recurrence is summarized in Table 4. Univariable predictors of AF recurrence were age, body mass index (BMI), hypertension, HAS-BLED score, GPALS, and LA stiffness as well as the presence of any LVA. Low GPALS and elevated LASI remained strong independent predictors of recurrent atrial tachyarrhythmias following CA procedures (Table 4) in multivariable analysis after correcting for the other clinical parameters.

**TABLE 4:** Uni- and multivariable Cox analysis for predictors of AF recurrence after CA.

	Univariable analysis		Multivariable model 1		Multivariable model 2	
			
	HR [95% CI]	*p*-Value	HR [95% CI]	*p*-Value	HR [95% CI]	*p*-Value
Age	1.03 [0.99; 1.07]	0.103	0.97 [0.92; 1.02]	0.237	1.00 [0.95; 1.05]	0.896
Sex	0.59 [0.30; 1.16]	0.130				
BMI	0.93 [0.86; 1.00]	0.050	0.91 [0.85; 0.99]	0.026	0.90 [0.83; 0.98]	0.026
Diabetes	0.94 [0.36; 2.40]	0.897				
Hypertension	1.92 [0.99; 3.72]	0.054	1.50 [0.58; 3.87]	0.402	1.31 [0.48; 3.54]	0.594
CHARGE-AF risk score	1.11 [0.82; 1.49]	0.483				
CAD	1.13 [0.51; 2.48]	0.761				
Pulmonary disease	0.80 [0.35; 1.81]	0.597				
eGFR	0.98 [0.96; 1.00]	0.185				
Loop diuretics	1.87 [0.96; 3.66]	0.065				
CHA2DS2-VASC score	1.10 [0.90; 1.33]	0.326				
HATCH score	1.03 [0.77; 1.37]	0.841				
APPLE score	1.09 [0.85; 1.39]	0.468				
HAS-BLED score	1.37 [1.03; 1.82]	0.031	1.13 [0.70; 1.82]	0.609	1.12 [0.70; 1.80]	0.616
Total # of episodes of AF	0.98 [0.80; 1.20]	0.883				
Any LVA	1.95 [1.0; 3.78]	0.04				
LVEF (%)	1.00 [0.97; 1.02]	0.995				
LV mass index	0.99 [0.97; 1.00]	0.192				
LAVI	1.00 [0.99; 1.02]	0.297				
E wave	4.21 [0.69; 25.6]	0.118				
E/e′	1.05 [0.95; 1.15]	0.334				
LAEF	0.98 [0.95; 1.00]	0.155				
FAC	0.95 [0.91; 1.00]	0.067				
GPALS	0.89 [0.78; 0.95]	0.003	0.85 [0.77; 0.95]	0.004		
LA Stiffness	1.34 [1.08; 1.65]	0.006			1.30 [1.03; 1.65]	0.026

*HR, hazard ratio; all others as Table 1.*

**FIGURE 4:**
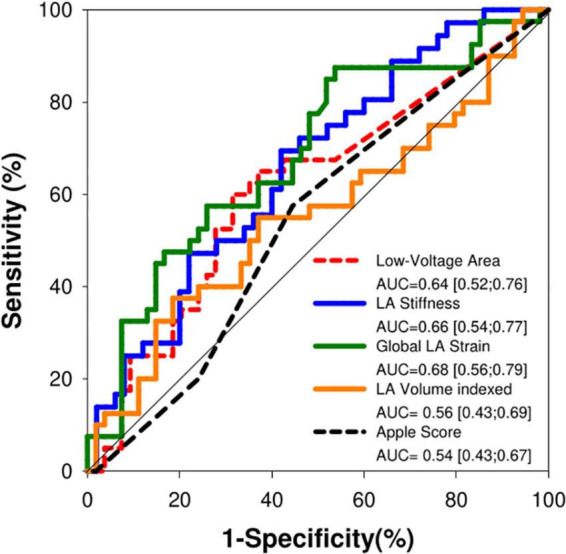
Receiver-operating characteristic curve analysis for prediction of atrial fibrillation recurrence after catheter ablation. AUC, area under the curve; *p* = 0.02 GPALS vs. Apple score and *p* = 0.05 GPALS vs. LAVI.

**FIGURE 5:**
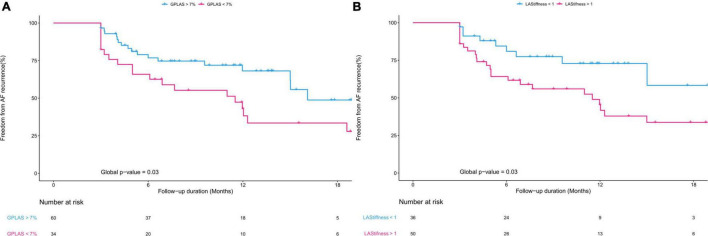
Kaplan–Meier graphs showing freedom from AF recurrence after CA according to GPALS **(A)** and LA stiffness **(B)**.

The ROC curves for prediction of AF recurrence after the CA procedure for the different echocardiographic parameters are shown in Figure 4 and Kaplan–Meier curves are shown in Figure 5. LA stiffness had the highest AUC (0.685) to predict AF recurrence, which was significantly higher (*p* = 0.02) than that of the Apple score (AUC 0.54) and LAVI (AUC 0.561, *p* = 0.05 vs. GPALS). Both GPALS and LA stiffness had similar (*p* = 0.23 and 0.07, respectively) good predictive accuracy to predict AF recurrence than LVA. Both GPALS and LA stiffness were able to significantly improve the c statistic to predict AF recurrence over CHARGE-AF (c score = 0.465, c score of GPALS + CHARGE-AF = 0.713, *p* < 0.001, c score of LA stiffness and CHARGE-AF = 0.686, *p* = 0.01), or CHA_2_DS_2_-VAS_c_ score (c score = 0.579, c score of GPALS and CHA_2_DS_2_-VAS_c_ = 0.687, *p* < 0.001, c score of LA stiffness and CHA_2_DS_2_-VAS_c_ = 0.685, *p* = 0.02). GPALS and LA stiffness also improved the NRI over the CHARGE-AF index (NRI 0.67, 95% CI [0.33–1.13] for GPALS and NRI 0.73, 95% CI [0.12–0.91] for LA stiffness, respectively), and over the CHA_2_DS_2_-VAS_c_ score (NRI 0.43, 95% CI [−0.14 to 0.69] for GPALS and NRI 0.52, 95% CI [0.10–0.84], respectively) for LA stiffness to predict AF recurrence at 9 months.

### Intraobserver and Interobserver Variability

Interobserver CV values were 10.2 ± 13.8% for LAVI, −0.5 ± 2.5% for GPALS, 4 ± 6% for E/e′ ratio, and 0.2 ± 0.2% for LA stiffness, respectively. Interobserver ICC were 0.98 [0.93; 0.99] for LAVI, 0.86 [0.45; 0.97] for GPALS, 0.95 [0.89; 0.98] for E/e′ ratio, and 0.98 [0.98; 0.99] for LA stiffness, respectively (all *p* < 0.001).

Intraobserver CV values were 3.2 ± 10.8% for LAVI, 0.4 ± 0.9% for GPALS, 3 ± 5% for E/e′, and 0.1 ± 0.3 for LA stiffness, respectively, and the corresponding ICC were 0.98 [0.93; 0.99], 0.96 [0.86; 0.99], 0.97 [0.93; 0.99], and 0.98 [0.94; 0.99], respectively (all *p* < 0.001).

## Discussion

We performed a non-invasive evaluation of LA structure and function acquired in patients while being in persistent AF before CA. The salient findings of our study were as follows:

1) LAVI, E/e′ GPALS, and LA stiffness during persistent AF independently allowed to predict LVA during electro-anatomical mapping. Of all the parameters, LA stiffness had the highest diagnostic accuracy for the prediction of LVA.
2) GPALS and LA stiffness are also able to predict the success of CA procedures and maintenance of SR with similar accuracy as LVA extent. For this purpose, the predictive accuracy of GPALS is significantly better than the APPLE score and LAVI.
3) These new echocardiographic parameters evaluated in AF had high reproducibility and a low intraobserver and interobserver variability.

To the best of our knowledge, this is the first study comparing the predictive value of strain with LVA in a homogeneous population of patients being in persistent AF at the time of analysis. Structural and functional atrial remodeling is a well-established hallmark involved in the development and progression of AF (3). It is strongly associated with the development of progressive atrial fibrosis, which will ultimately lead to AF persistence. In the present work, we compared different non-invasive structural and functional parameters of LA remodeling to EAM-derived LVA extent as the reference method for evaluation of atrial fibrosis. EAM evaluates atrial voltage with predefined thresholds to identify LVA associated with the atrial scar. This widely used electrophysiological technique for atrial scar quantification has been validated against histopathology (20) and was found to have comparable results to the LGE-cMR based atrial scar quantification both in SR (21–23) and in AF (24, 25). LA dilatation is a well-known parameter associated with atrial remodeling severity before and during AF and was shown to be associated with poorer ablation outcomes and higher recurrence rates (26). Our study confirmed these findings to be associated with LVA. Moreover, the E/e′ ratio, a parameter indicative of increased LV filling, and hence of increased LA pressures, was associated with larger regions of LVA. This causal relationship is probably explained by the increased LA pressure and wall stress leading to structural remodeling and reduced atrial pump function (27). Similar findings of E/e′ ratio correlating with atrial fibrosis have been already illustrated, however, only in SR (16, 28). Our results corroborated that the association between LA pressure estimated by E/e′ and LA remodeling evaluated by LVA extent also remains valid in the setting of AF. In our study, the prediction of LVA was even more accurate for GPALS and LA stiffness. In SR, LA strain has three components: the reservoir function, the conduit function, and the contractile function (booster pump). Several studies have shown that the atrial booster pump and reservoir function has an important prognostic role in the prediction of AF occurrence (12, 29). In patients in SR, a relationship between the extent of LVA and LA strain and LA emptying fraction was reported (30). In AF, however, booster pump strain is absent, and only GPALS, a surrogate of LA conduit/reservoir function, can be assessed. GPALS by STE in SR was shown to inversely correlate with atrial fibrosis by histopathology in patients with mitral valve regurgitation undergoing valve surgery (31) and also in patients with advanced heart failure undergoing heart transplantation (32). To our knowledge, only a few studies evaluated the relation of LA strain to predict fibrosis during AF. Kuppahally et al. (12) found LA strain and strain rate to be inversely related to LA wall fibrosis, by LGE-cMR in patients with paroxysmal or persistent AF, but in this cohort, a minority (29%) of patients were studied while in AF. LA stiffness is another parameter of interest but has been less evaluated. It is computed as the ratio of E/e′ to LA peak strain (19). It thus represents an estimate of LA compliance estimated from two non-invasive measurements of LA deformation (strain) and atrial pressure (estimated from E/e′ ratio). Indeed, E/e′—the ratio of the early diastolic velocity of the mitral inflow to early diastolic velocity of the mitral annulus provides a close approximation of LV filling pressures in a wide spectrum of diseases. At the time of mitral valve opening, peak LA pressure is equal to LV filling pressure, and thus, E/e′ can be considered as a substitute for LA peak pressure. Kishima et al. (17) demonstrated a relation between LA stiffness and LVA, however, only in patients with paroxysmal AF who underwent STE while in SR. In this context, the principal novelty of our study is the demonstration of the usefulness of the different parameters to predict atrial fibrosis by LVA while being in AF.

Another important finding of our study was the demonstration that GPALS and atrial stiffness can predict the success of CA. It has been well demonstrated that atrial fibrosis estimated by LGE-cMR (33) or LVA (10, 11) predicts an increased risk of post-ablation arrhythmia recurrence. In our present study, clinical risk scores, such as the CHARGE-AF CHA_2_DS_2_-VAS_c_, HATCH, or APPLE, were predictive of arrhythmia recurrence, and the only clinical score associated with arrhythmia recurrence was the HAS-BLED score. As expected, advanced age, increased BMI, history of systolic arterial hypertension, and the degree of atrial fibrosis estimated by LVA predicted the risk of AF recurrence. However, the estimation of atrial function by GPALS and LA stiffness had a similar good predictive value as LVA. While other studies had already linked parameters, such as LAVI, E/e′ GPALS, and atrial stiffness with AF recurrence, this was mostly shown in patients with paroxysmal AF during SR (14–18).

### Clinical Implications

The success rate of CA in persistent AF is lower than in paroxysmal AF and is determined by the duration of AF and the degree of LA structural remodeling. Therefore, for the decision of AF CA, it is recommended to consider the risk of AF recurrence (1). Whereas EAM-LVA is a straightforward procedure for the electrophysiologist, providing rapid information on structural remodeling, this test has little usefulness for the selection of patients, as it is invasive and can only be performed when the decision of CA has already been taken. LGE-cMR is currently the only well-validated non-invasive technique to evaluate LA fibrosis; however, it is time-consuming, has limited accessibility, and the reproducibility of exact quantification of the degree of fibrosis is not standardized. This restricts its clinical use to expert centers. Echocardiography is more readily available and less expensive. We showed that the GPALS and atrial stiffness measurements have high interobserver and intraobserver reproducibility and that their predictive value for AF recurrence was non-inferior to LVA. This suggests that this simple test could be useful for the preoperative selection of candidates for CA.

### Limitations

This was a single-center study with limited clinical follow-up. Our LVA measurements might not be applicable to other mapping systems and catheters. While all echocardiography exams were performed in AF, EAM was performed both in AF and in SR. It was however demonstrated that LVA measurements between AF and SR correlate well when a lower voltage cutoff is used in AF (34, 35), as we did in our present study. Echocardiographic acquisitions were performed from an average of only two cardiac cycles. Averaging more cycles might have lowered the beat-to-beat variability of measurements in AF. TTE was analyzed in duplicate by two experienced blinded echocardiographers (SMA and QG). The researchers were not independent. These results should be confirmed using other vendors because of the inter-vendor variability. We did not use long-term ECG monitoring, and therefore, we might have not been able to detect all asymptomatic episodes of AF during follow-up. Our findings should therefore also be confirmed with long-term arrhythmia monitoring. Finally, we did not perform LGE-cMR, and therefore, we could not evaluate whether STE measurements would have had better predictive accuracy to predict CA success than this technique.

## Conclusion

In subjects with persistent AF, there is a significant correlation between indexed LA volume, GPALS, the LA pressure estimated by E/e′ ratio, and LA stiffness – acquired during AF – and the degree of LVA. In addition, low GPALS and elevated LA stiffness predicted a higher recurrence rate of atrial tachyarrhythmias after the index CA procedures. This supports the use of STE to non-invasively predict the severity of atrial fibrosis and the success of CA procedures in patients with persistent AF.

## Data Availability Statement

The raw data supporting the conclusions of this article will be made available by the authors, without undue reservation.

## Ethics Statement

The studies involving human participants were reviewed and approved by the Comite Ethique Hospitalo Facultaire de l’Université de Louvain. The patients/participants provided their written informed consent to participate in this study.

## Author Contributions

SM contributed to study design, data acquisition, and statistical analysis, and drafted and approved the manuscript. QG, CS, VV, and AW contributed to data acquisition and manuscript revision, and read and approved the submitted version. J-BP contributed to study design and manuscript revision, and read and approved the submitted version. CB contributed to manuscript revision, and read and approved the submitted version. VR contributed to study design, data acquisition, and manuscript revision, and read and approved the submitted version. BG contributed to study design, statistical analysis and manuscript revision, and read and approved the submitted version. All authors contributed to the article and approved the submitted version.

## Conflict of Interest

The authors declare that the research was conducted in the absence of any commercial or financial relationships that could be construed as a potential conflict of interest.

## Publisher’s Note

All claims expressed in this article are solely those of the authors and do not necessarily represent those of their affiliated organizations, or those of the publisher, the editors and the reviewers. Any product that may be evaluated in this article, or claim that may be made by its manufacturer, is not guaranteed or endorsed by the publisher.
